# Comparative Effect of Aqueous and Methanolic Bupleuri Radix Extracts on Hepatic Uptake of High-Density Lipoprotein and Identification of the Potential Target in HFD-Fed Mice

**DOI:** 10.1155/2019/9074289

**Published:** 2019-12-05

**Authors:** Zhibin Tan, Yijia Huang, Fenglian Chen, Cheng Cao, Shuling Wang

**Affiliations:** ^1^School of Chinese Pharmaceutical Science, Guangzhou University of Chinese Medicine, Guangzhou 510006, China; ^2^Shunde Hospital of Guangzhou University of Chinese Medicine, Foshan 528000, China

## Abstract

Our previous study found saikosaponin b_2_ (SSb_2_) increased high-density lipoprotein (HDL) uptake in HepG2 cells. SSb_2_ is only found in aqueous Bupleuri Radix extract, and it is one of the secondary saponins derived from saikosaponin d (SSd), which exists in the methanolic extract. This study aimed to compare the effect of aqueous extract of Bupleuri Radix on hepatic uptake of HDL with methanolic extract and to reveal the underlying mechanism of enhancing HDL uptake in mice fed with high-fat diet (HFD). Cellular HDL uptake in each group was quantified by flow cytometry. Bioactive components bound to the HepG2 cytomembrane were detected with HPLC-DAD. RNA sequencing was performed to screen the underlying target on hepatic HDL-uptake, and western blotting was conducted to verify differential protein expression. Significant increases of HDL uptake by HepG2 cells were observed in all groups of aqueous extract of Bupleuri Radix, while no effect or negative effect was observed in the methanolic extract. Saikosaponin b_1_ (SSb_1_) and SSb_2_ were detected in the desorption elute of the aqueous extract from the HepG2 cytomembrane, while saikosaponin a (SSa) and SSd were not found. Remarkable upregulation of FGF21 in HFD-fed mice liver was affirmed after treatment with aqueous extract. This study suggested that aqueous Bupleuri Radix extract could promote hepatic HDL uptake in vitro but methanolic extract could not, and FGF21 might be the potential target.

## 1. Introduction

Bupleuri Radix, the root of *Bupleurum chinense* DC. *or Bupleurum scorzonerifolium* Willd. [1], is a widely used herbal medicine in China, which regulates functions of the internal organs to relieve fever, disperse the stagnation of liver-qi, and uplift yang-qi. Because of its liver tropism [2], according to the theory of traditional Chinese medicine, Bupleuri Radix is broadly applied in the development of therapeutic strategies for liver diseases [3], e.g., hepatitis [4], hepatoma [5], hepatocellular carcinoma [6], and hyperlipemia-related diseases [7]. Saikosaponins in majority composed effective components of Bupleuri Radix, and to date, more than 100 saikosaponins have been isolated and identified, including primary saikosaponins and secondary saikosaponins, such as saikosaponin a (SSa), b_1_ (SSb_1_), b_2_ (SSb_2_), d (SSd), and others, among which SSa and SSd are believed to be the most bioactive ingredients [8].

SSa and SSd are characteristic components in crude plant material of Bupleuri Radix, and they are configurational isomers and have an oxygen ether ring in common, which is unstable and splits during decocting process with boiling water, triggering structure transformation into secondary saikosaponins, SSb_1_ and SSb_2,_ respectively. Consequently, in aqueous extract of Bupleuri Radix, SSd is hardly detected and a mass of SSb_2_ is produced instead and the transformation occurs much more in acid water as reported in our previous study [9] or another study [10]. As known to all, the conventional extraction of Chinese medicinal herbs is decocting with boiling water, and of note, crude plant material of Bupleuri Radix needs to be processed with vinegar [11] before used for liver diseases as prescribed in ancient documents of Chinese traditional medicine, but with the innovation of modernization and internationalization of Chinese herbal medicines, ethanol extraction is vastly applied in the production. From previous studies, it is obvious that the composition of saikosaponins is extremely different between the aqueous and methanolic extract of Bupleuri Radix, primary saikosaponins mainly in the methanolic extract and secondary saikosaponins in the aqueous extract. Whether primary or secondary saikosaponins are active ingredients in the Bupleuri Radix extract is unclear as yet. The high biological activity of saikosaponins makes these compounds promising agents for use in medicine, particularly as antihepatopathy drugs [4, 8]. Our pioneer trial found saikosaponin b_2_ promoted hepatic uptake of HDL in vitro [12]. The aim of this study was to evaluate and compare the effects of aqueous and methanolic extracts of Bupleuri Radix on hepatic uptake of HDL and also to explain its underlying mechanisms of regulating lipid metabolism.

## 2. Materials and Methods

### 2.1. Materials and Chemicals

Bupleuri Radix (lot no. 120601) was purchased from Guangzhou Chinese Medicine Corporation and identified, and Professor Haibo Huang in the Guangzhou University of Chinese Medicine verified the identity of the plant materials as *Bupleurum chinense* DC. A culture flask was bought from Corning Costar Corporation (Cambridge, MA, USA). 0.25% trypsin-EDTA was purchased from GIBCO (Grand Island, NY, USA). Dimethyl sulfoxide (DMSO) was obtained from Sigma (St. Louis, MO, USA). Dulbecco's modified eagle medium (DMEM) and phosphate-buffered saline (PBS, pH 7.4) were obtained from GIBCO (Grand Island, NY, USA). Fetal bovine serum was acquired from Hyclone (Logan, UT, USA). 1,1-Dioctadecyl-3,3,3,3-tetramethylindocarbocyanine perchlorate- (DiI-) labeled human HDL (DiI-HDL) was obtained from Guangzhou Yiyuan Biotechnology Co., Ltd. (Guangzhou, China). Purified water was prepared with a Milli-Q system (Millipore, Milford, MA, USA). Saikosaponin a, saikosaponin d, saikosaponin b_1,_ and saikosaponin b_2_ were bought from Chengdu Pufei De Biotech Co., Ltd. (Chengdu, China), and the purity is above 98%. All other reagents used were of analytical grade in the present study.

### 2.2. Preparation of Bupleuri Radix Extracts

Both aqueous and methanolic extracts of Bupleuri Radix were obtained by the method of reflux extraction with subsequent column chromatographic purification as previously described [12]. In brief, samples of dried Bupleuri Radix were pulverized into powder with an average particle size of 250 *μ*m, weighed as 20.0 g, soaked in 200 mL extractive solvent (water or 8% ammoniac methanol) for 30 min, and then refluxed in a boiling water bath for 1 h. Both extracts were concentrated and dried under 40°C vacuum, and the residues were redissolved in 50.0 mL water, respectively. 25 mL water solutions were centrifuged at 5000 rpm for 10 min, and the supernatants were passed through a 2 × 30 cm column of polyamide (8 g, SI JIA Biochemical Plastic, Zhejiang, China). The columns were rinsed with 25 mL distilled water, 25 mL 20% ethanol, and 50 mL 40% ethanol successively at a flow rate of 2 BV/h. BV refers to the volume of solution equivalent to the resin volume in place. The eluates of 40% ethanol were harvested and concentrated to dryness at 40°C under vacuum. The residues were redissolved in 10 mL of HPLC-grade methanol at a concentration of 2.0 g crude drug per milliliter, and before HPLC analysis each sample was filtered through a 0.22 *μ*m microfiltration membrane.

### 2.3. High-Performance Liquid Chromatography Profiling of Bupleuri Radix Extracts

A characteristic fingerprint method using high-performance liquid chromatography with photodiode array detection (HPLC-DAD) was developed to profile and compare the components in aqueous and methanolic extraction of Bupleuri Radix and also for quality control of the extract samples [13]. Analysis was performed on a Shimadzu high-performance liquid chromatography system equipped with an LC-20AT pump, a vacuum degasser, an SIL-20A automatic sampler, a CTO-10AS VP column oven, and an SPD-M20A detector connected to LC Solution software. The mobile phase was filtered through a 0.45 *μ*m membrane filter and degassed with a vacuum degasser. The autosampler rinse solution was methanol. Chromatographic separation was carried out on an octadecylsilyl analytical column (LUBEX Ecosil, 250 × 4.6 mm i.d., 5 *μ*m) with water and acetonitrile as the mobile phases at a flow rate of 1.0 mL/min. Column temperature was maintained at 30°C, and total run time was 75.0 min. Gradient elution was used as follows: 0 min 15% acetonitrile, 15 min 25% acetonitrile, 55 min 40% acetonitrile, and 75 min 55% acetonitrile. The eluate from the analytical column was monitored at UV absorption wavelength of 204 nm and 254 nm for primary and secondary saikosaponins, respectively.

### 2.4. Cytotoxicity Assessment of Bupleuri Radix Extracts

Cell viability was assessed to determine the cytotoxicity of Bupleuri Radix extracts using a Cell Counting Kit-8 (CCK-8) colorimetric assay. HepG2 cells were obtained from the Shanghai Institute of Cell Biology (Shanghai, China) and cultured in DMEM containing 10% fetal bovine serum (v/v) and supplemented with 100 U/mL penicillin and 100 *μ*g/mL streptomycin. Bupleuri Radix extracts were dried with a nitrogen sample concentrator (UGC-24W, Beijing Yousheng Union Technology, China) and redissolved to make a series of concentrations in DMEM with 0.1% DMSO as cosolvent, all sterilized by 0.22 *μ*m aseptic filters. HepG2 cells in the logarithmic growth phase were inoculated into each well at a density of 5 × 10^3^ cells/well and incubated at 37°C for 24 h to attach to the surface of 96-well plates prior to Bupleuri Radix extracts exposure. Cells were treated with a series of concentrations of sterilized aqueous or methanolic extract of Bupleuri Radix (5, 10, 15, 20, 40, 80, and 100 mg/mL or 5, 10, 15, 20, 30, 35, and 40 mg/mL, respectively) and then incubated at 37°C for 24 h. Control cells were exposed to 0.1% DMSO in the culture medium. At the end of the treatment period, the culture medium was replaced with fresh medium containing 10 *μ*L CCK-8 solution and incubated at 37°C for 1 h until the development of purple formazan. The formazan product was solubilized in isopropanol. Next, the plates were vibrated in a microplate oscillator at low speed for 10 min. The absorbance was measured at 450 nm with a microplate reader (Thermo Fisher 1510, Thermo Fisher Scientific, USA). The inhibition rate was shown as relative percentages compared to the control groups and calculated as (control group−tested group)/control group × 100%. All tests were performed in triplicate. The replicates were averaged to calculate the IC_50_ value and cell survival percentages.

### 2.5. DiI-HDL Uptake Assay

To evaluate hepatic uptake of HDL of Bupleuri Radix extracts, we utilized purified high-density lipoproteins labeled with the fluorescent probe 1,1′-dioctadecyl-3,3,3′,3′-tetramethylindocarbocyanine perchlorate (DiI-HDL). DiI is a fluorescent dye that when excited with wavelengths around 514 nm shows an emission peak in the range of 550 nm, suitable for viewing with a common rhodamine filter. Both aqueous and methanolic extracts of Bupleuri Radix were dried with a nitrogen sample concentrator (UGC-24W, Beijing Yousheng Union Technology, China) and redissolved to make low, medium, and high dose in DMEM with 0.1% DMSO as cosolvent, all sterilized by 0.22 *μ*m aseptic filters. The administered doses were set according to the results of drug cytotoxicity in CCK-8 tests to ensure they were safe for the viability of HepG2 cell lines. In particular, HepG2 cells were seeded in 24-well plates at a concentration of 5.0 × 10^4^ cells/well and incubated at 37°C and 5% CO_2_ for 24 h. After washed twice with PBS, the cells were treated with low, medium, or high dose of Bupleuri Radix extract in the Dulbecco's modified eagle medium. Following administration, the cells were cultured for 24 h before 2 *μ*g/mL DiI-HDL was added, and subsequently the cells were cultured for an additional 5 h at 37°C. After cell harvest and washes, fluorescence measurements were performed with a flow cytometer (FACSCalibur, Becton Dickinson, USA) as described previously [12]. Data were analysed by the Flowjo software version 7.6.1 (Tree Star Inc., Ashland, Oregon). For visualization, cells were observed under an upright fluorescent microscope (Olympus BX53, Olympus Corporation, Japan), and microphotographic camera images were acquired at 40 magnification (Bio Rad, USA). In the time-lapse experiment, HepG2 cells were plated in a 6-well plate (Corning) at a density of 10 × 10^4^ cells/well and incubated for 24 h. 10 *μ*g DiI-HDL were added in each well, and IncuCyte ZOOM real-time imaging platform was utilised to record the vital uptake of DiI-HDL by HepG2 cells at one spot for up to 5 h. Imaging was taken every hour using a 20x objective lens and then analysed using the IncuCyte™ Basic Software. Green channel acquisition time was 400 ms.

### 2.6. Membrane Immobilized Chromatography

HepG2 cells were plated into a cell culture flask (75 cm^2^) and incubated with aqueous or methanolic Bupleuri Radix extract in a humidified incubator under 5% CO_2_ at 37°C for 2 h. Cell membrane extraction, sample preparation, and HPLC analysis were described in detail in our previous study [11]. Briefly, citric acid-disodium hydrogen phosphate buffer (pH = 4.0) was prepared consisting of 0.11 M citric acid and 0.5 M disodium hydrogen phosphate. HepG2 cells were seeded into a cell culture flask (25 cm^2^) and cultured in a humidified incubator with 5% carbon dioxide (CO_2_) at 37°C until they reached confluence (about 2 days after seeding). Aqueous and methanolic extracts diluted with DMEM (high glucose) into 60 mg/mL and 30 mg/mL, respectively, were added to prepared HepG2 cells and incubated for 2 h. Remove the culture solution, wash the flask with 4 mL PBS nine times, and subsequently centrifuge at 1000 rpm for 5 min to remove the possible nonselectively combining components. The first and ninth eluates were collected as contrast for HPLC analysis. Lastly, the cells were denatured and extracted with 2 mL of citric acid-disodium hydrogen phosphate buffer (pH = 4.0) and cultured in a humidified incubator with 5% carbon dioxide (CO_2_) at 37°C for 1 h and the supernatant was harvested as a desorption solution after centrifuged at 10,000 rpm for 10 min. The PBS eluates and desorption solution were evaporated at 40°C under vacuum. The residues were redissolved in 100 *μ*L of methanol and centrifuged at 13,000 rpm for 10 min to get the supernatant for LC-Q-TOF/MS/MS analysis. Chromatographic separation was performed on an ultraperformance liquid chromatography system (Nexera X2, Shimadzu, Japan) equipped with an LC-30AD pump, a vacuum degasser, an SIL-30AC automatic sampler, and a CTO-20AC column oven connected to Analyst TF software. Cell lysate was analysed on an octadecylsilyl analytical column (LUBEX Ecosil, 250 × 4.6 mm i.d., 5 *μ*m) with water and acetonitrile as the mobile phases at a flow rate of 1.0 mL/min by LC-Q-TOF/MS/MS (Triple TOF 5600, AB SCIEX, USA) with the scan type of TOF MS, and ionization was achieved using Duo Spray ESI in the negative mode. Column temperature was maintained at 25°C, and injection volume was 20 *μ*L. Gradient elution was used as follows: 0 min 15% acetonitrile, 15 min 25% acetonitrile, 55 min 40% acetonitrile, and 75 min 55% acetonitrile. MS instrument parameters were set as follows: atomization gas 55 psi, auxiliary gas 55 psi, gas curtain 35 psi, atomization temperature 550°C, ionization potential 4500 V, declustering potential 100 V, and collision energy 10 eV. Method validation tests including calibration curve, instrumental precision test, repeatability test, robustness test, and recovery test were performed during the development of analytical procedure.

### 2.7. Influence of Bupleuri Radix Extract on Liver Tissues of HFD-Fed Mice

To provide insights into mechanisms of upregulating HDL uptake, RNA-sequencing and histological analysis were conducted on liver tissues from four biological replicates. Male C57BL/6 mice (8 weeks old, weighing 18–22 g) were randomly divided into 3 groups: Chow-, HFD-, and HFD/Bupl-mice (*n* = 4 in each group). All mice were acclimatized for 1 week with adequate food and water. Chow-mice were fed with chow and HFD- and HFD/Bupl-mice were fed with high-fat diet for 20 weeks. From the 16th week, HFD/Bupl-mice were treated with aqueous Bupleuri Radix extract for 4 weeks and Chow- and HFD-mice were given the same amount of distilled water. One hour after the last administration, liver tissues were dissected after perfusion. Histological studies were performed on 5 *μ*m-thick sections of formalin-fixed and paraffin-embedded liver tissues. Paraffin sections were stained with hematoxylin-eosin (HE) and observed under an optical microscope. RNA-sequencing analysis on liver tissues was conducted by the Beijing Genomics Institute. Total RNAs were converted into cDNA libraries as templates for high-throughput sequencing using Illumina HiSeq 2000 following the Illumina TruSeq RNA sample preparation protocol. In brief, the first-strand cDNA was synthesized from 5 *μ*g of total RNA using oligo-dT primers and subsequently converted into blunt ends via exonuclease/polymerase. After adenylation of 3′ ends of DNA fragments and ligating, cDNA fragments were enriched using PCR reaction. The established cDNA libraries were verified with Agilent 2100 Bioanalyzer and ABI StepOnePlus Real-Time PCR System. After quality control of raw reads, clean reads were generated and analysed by alignment using the Burrows–Wheeler transform and Bowtie 2.

### 2.8. Western Blotting Test for Validation of Potential Target

Samples from each group were collected in RIPA lysis buffer (50 mM Tris-Cl [pH 7.4], 1% Triton X-100, 1% sodium deoxycholate, 0.1% sodium dodecyl sulfate [SDS], and 150 mM NaCl), and a protease inhibitor tablet was added (Beyotime Sciences, USA). The samples were centrifuged at 12,000 rpm at 4°C, and protein concentration was determined using a BCA protein assay (CwBio Sciences, CN). Lysates were separated using sodium dodecyl sulfate-polyacrylamide gel electrophoresis (SDS-PAGE) and transferred to polyvinylidene difluoride membranes. The membrane was probed with *β*-actin horseradish peroxidase (HRP) (Proteintech, CN) and blocked with 5% BSA before incubation with the primary rabbit anti-mouse FGF21 antibody (Abcam, UK). An HRP-conjugated goat anti-rabbit IgG antibody (Millipore) was applied, and after incubation, the membrane was developed with an Amersham-enhanced chemiluminescence western blotting detection system (Bio-Rad, Versa Doc 4000MP, USA).

### 2.9. Statistical Analysis

All statistical analyses were performed using GraphPad Prism 6.0 (GraphPad Software, Inc., San Diego, CA). Experimental data were obtained by three independent experiments and expressed as mean ± standard deviation (SD). *p* values of less than 0.05 were considered statistically significant.

## 3. Results and Discussion

### 3.1. Characterization of Major Components in Bupleuri Radix Extracts

**Figure 1:**
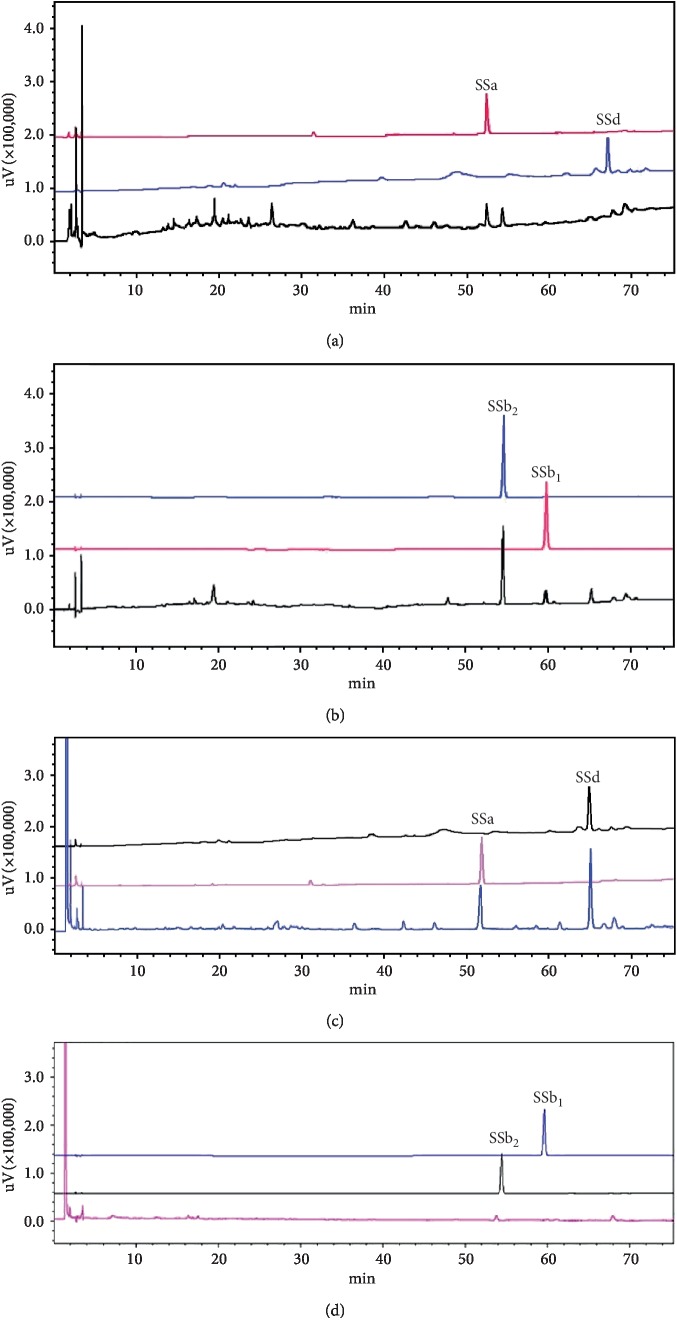
HPLC-DAD chromatograms of Bupleuri Radix extracts. Aqueous extract detected at wavelength 204 nm (a) and 254 nm (b). Methanolic extract detected at wavelength 204 nm (c) and 254 nm (d). SSa, saikosaponin a; SSd, saikosaponin d; SSb_2_, saikosaponin b_2_; SSb1, saikosaponin b_1_.

A rapid and simple chromatographic fingerprint method was established to evaluate and control the quality of Bupleuri Radix extracts by HPLC-DAD, by which four major constituents, SSa, SSb_1_, SSb_2,_ and SSd, were simultaneously detected in samples of Bupleuri Radix extracts. The standard samples of of SSb_1_ (0.3880 mg/mL), SSb_2_ (0.4032 mg/mL), SSa(0.4120 mg/mL), and SSd (0.3540 mg/mL) were prepared by dissolving suitable quantities of the standard substance in methanol. Because of the conjugated group of heterocyclic diene within their molecules, SSb_2_ and SSb_1_ display prominent characteristic ultraviolet absorption spectra with the maximum wavelength near 254 nm, while SSa and SSd as like most saturated saponins do not demonstrate characteristic absorption peaks in the region of spectrum (200–400 nm); therefore, the PDA detector was operated at 204 nm for SSa and SSd and 254 nm for SSb_1_ and SSb_2_. The HPLC-DAD fingerprint method permitted accurate and precise determination of SSa, SSb_1_, SSb_2,_ and SSd in the extraction of Bupleuri Radix. The chromatograms demonstrated the discrimination of aqueous and methanolic extraction of Bupleuri Radix: aqueous extract mainly comprised SSb_1_, SSb_2_, and a small amount of SSa (Figures 1(a) and 1(b)), whereas SSa and SSd were the major constituents in methanolic extract (Figures 1(c) and 1(d)).

### 3.2. Determination of Safe Dosage of Bupleuri Radix Extracts for HepG2 Cell Lines

**Figure 2:**
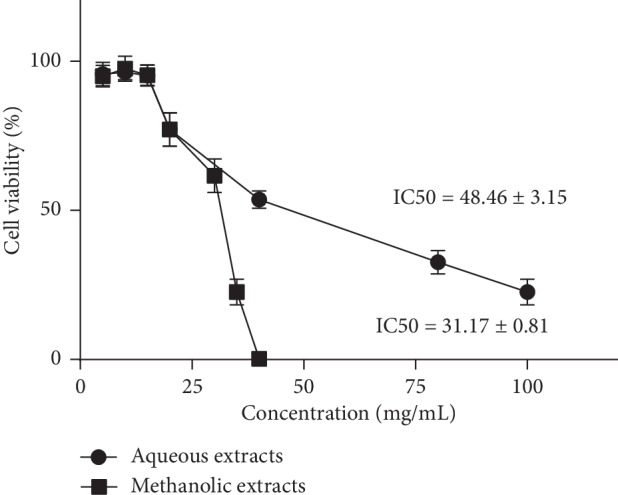
Evaluation of the toxicity of Bupleuri Radix extracts on HepG2 cells (*α* = 0.05).

Cell viability was assessed using a CCK-8 colorimetric assay. As shown in Figure 2, the cell viability was obviously inhibited when treated with aqueous extract at a concentration over 40 mg/mL or methanolic extract over 30 mg/mL. The CCK-8 tests established the 50% inhibiting concentration (IC_50_) dose of aqueous extract toward HepG2 cell lines to be 48.46 ± 3.15 mg/mL and methanolic extract to be 31.17 ± 0.81 mg/mL. However, it was also clearly observed that either aqueous or methanolic extract had no influence on the viability of HepG2 cells at concentrations of 5, 10, and 15 mg/mL after culturing for 24 h, compared with the negative control group. These findings indicated that Bupleuri Radix extracts exhibited cytotoxicity against HepG2 cell lines in a concentration-dependent manner, but they could be safely administered to cells at a low concentration of 5, 10, or 15 mg/mL. Based on these results, the safe concentrations of 5, 10, and 15 mg/mL were used in HDL uptake experiments, respectively, designated as low, medium, and high dosage for both aqueous and methanolic extracts.

### 3.3. Flow Cytometry, Microscopy, and Real-Time Imaging of DiI-HDL Uptake In Vitro

**Figure 3:**
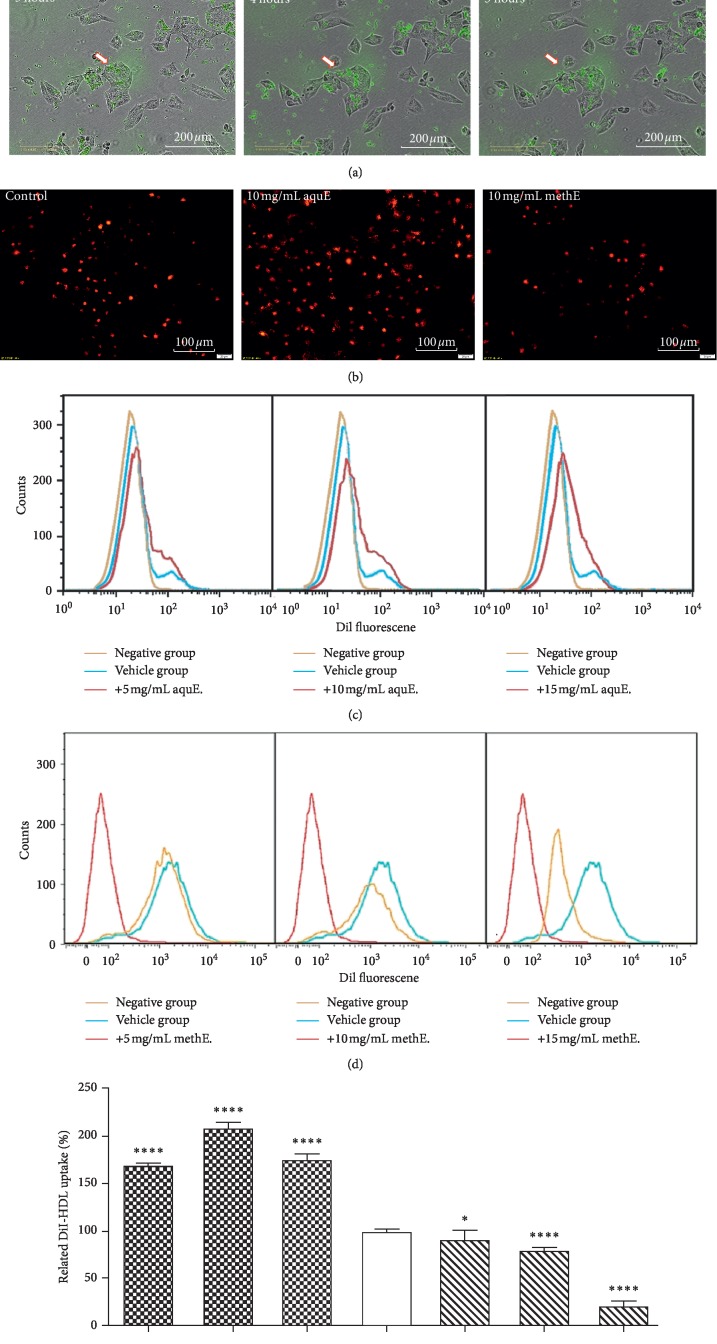
Flow cytometry, microscopy, and real-time imaging of DiI-HDL uptake by HepG2 cells. (a) The real-time imaging station IncuCyte ZOOM recorded HDL uptake by HepG2 cells at 0 h, 1 h, 2 h, 3 h, 4 h, and 5 h after exposure to DiI-HDL. (b) Fluorescent images of DiI-HDL uptake (40x) were obtained by Olympus BX53 after 24 hours of exposure to 10 mg/mL aqueous extract or methanolic extract of Bupleuri Radix. (c) FACS histograms of the fluorescence signal related to DiI-HDL uptake in 24 h cultured fibroblasts after treated with 5 mg/mL, 10 mg/mL, or 15 mg/mL aqueous Bupleuri Radix extract, respectively, from the left to right. (d) FACS histograms of the fluorescence signal related to DiI-HDL uptake in 24 h cultured fibroblasts after treated with 5 mg/mL, 10 mg/mL, or 15 mg/mL methanolic Bupleuri Radix extract, respectively, from the left to right. (e) The aqueous extract induced drastic increase of HDL uptake in vitro, while the methanolic extract reduced it. ^*∗*^*p*=0.05; ^*∗∗∗∗*^*p* < 0.0001 compared to the control group (*t*-Student test, unpaired two-tailed). Cells were incubated with DiI-HDL for 5 h before the harvesting times and prepared for FACS analysis as reported in Section 2. Each experiment was carried out at least twice in triplicate. At least 10000 cells for each group were analysed.

The real-time images taken on the IncuCyte ZOOM real-time imaging platform illustrated the particles of DiI-HDL phagocytized by HepG2 cells (Figure 3(a)), and a trend of progressive increase of HDL uptake over time was observed. Uptake of DiI-HDL was determined by fluorescence-activated cell sorting (FACS) and expressed as the percentage of HepG2 cells that took up DiI-HDL. Compared to the control cells (Figure 3(e)), DiI-HDL uptake by HepG2 cells incubated with 5, 10, or 15 mg/mL aqueous extract was significantly enhanced and the uptake percentage was 168.8 ± 1.4, 207.6 ± 4.3, and 173.3 ± 4.7, respectively (*p* < 0.001, *n* = 10,000 cells per sample), whereas approximately 10%, 20%, or 80% decrease of HDL uptake was observed following incubation with 5, 10, or 15 mg/mL methanolic extract, respectively (percentage uptake was 91.0 ± 3.7, 79.6 ± 1.2 and 20.5 ± 2.3, correspondingly). Consistent with FACS results, fluorescent microphotographs displayed parallel trends of HDL uptake by HepG2 cells (Figure 3(b)). These findings reflected a fact that secondary saikosaponins (e.g., SSb_1_ and SSb_2_) in aqueous extract of Bupleuri Radix were bioactive ingredients for promoting hepatic HDL uptake, whereas primary saikosaponins (e.g., SSa and SSd) in methanolic extract reduced HDL uptake.

### 3.4. Screening of Bioactive Components from HepG2 Cells Treated with Extract

**Figure 4:**
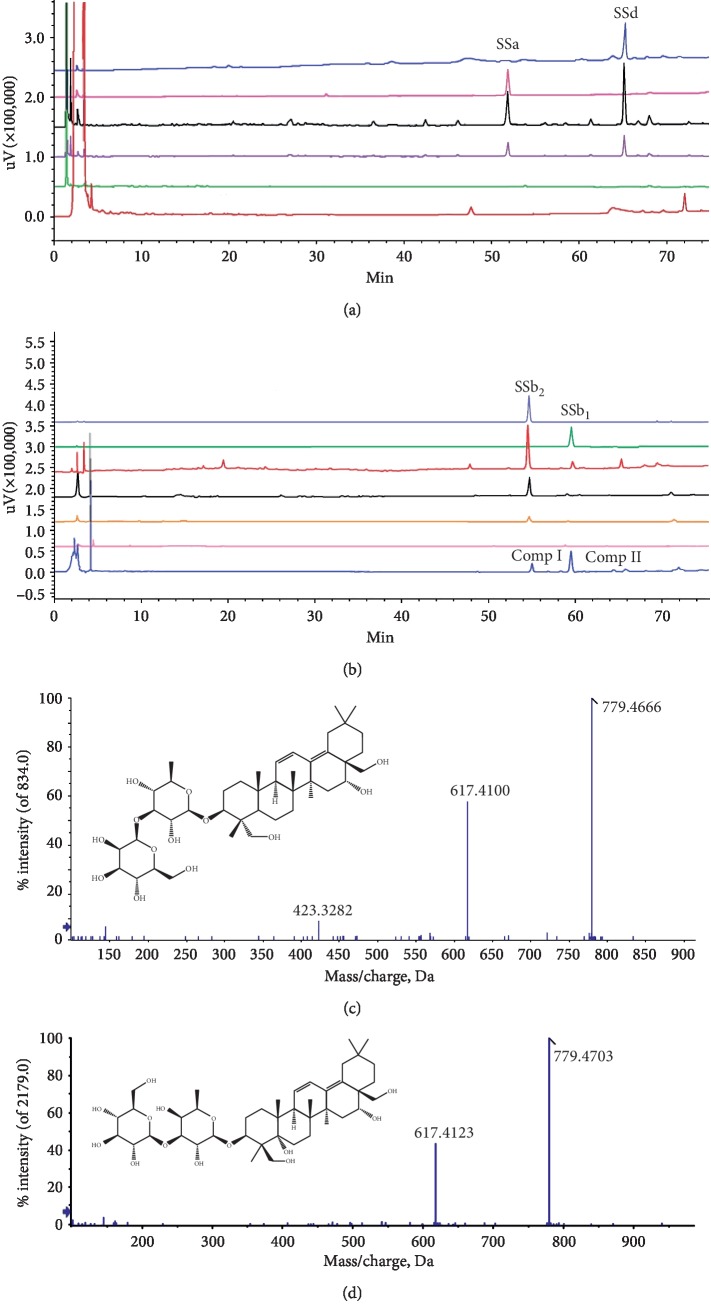
Screening the bioactive ingredients in Bupleuri Radix extract by membrane immobilized chromatography. (a) Superposed HPLC-DAD chromatograms of SSd standard, SSa standard, Bupleuri Radix aqueous extract, first PBS elution, ninth PBS elution, and desorption solution in sequence from the top to bottom detected at wavelength 204 nm. No compounds were found in the desorption solution. (b) Superposed HPLC-DAD chromatograms of SSb_2_ standard, SSb_1_ standard, Bupleuri Radix aqueous extract, first PBS elution, second PBS elution, ninth PBS elution, and desorption solution in sequence from the top to bottom detected at wavelength 254 nm. Compound I and II were found in the desorption solution of the HepG2 cell membrane, which showed the same retention time with standards SSb_2_ and SSb_1,_ respectively. (c, d) Secondary ion current chromatogram of LC-Q-Tof/MS/MS verified compound I was SSb_2_ and compound II was SSb_1_.

Basing on the hypothesis that combining with some receptors or channels on the cell membrane is the first step of drug action [14], HepG2 cell membranes were extracted to screen potential active components from Bupleuri Radix extracts. Two compounds were detected in the lysate of HepG2 cells exposed to aqueous extract of Bupleuri Radix and identified as SSb_1_ and SSb_2_ by LC-Q-TOF/MS/MS (Figures 4(c) and 4(d)), which were not found in the final desorption eluate. SSb_1_ and SSb_2_ were the major ingredients of the aqueous extract of Bupleuri Radix. Nevertheless, in the lysate of HepG2 cells exposed to the methanolic extract of Bupleuri Radix, its major ingredients SSa and SSd were not found, indicating they could not interact with the HepG2 cell membrane, which was corresponded with former FACS results of HDL uptake. From these data, SSb_1_ and SSb_2_ were anticipated to be potential active candidates in the Bupleuri Radix extract [2, 15].

### 3.5. FGF21 Was Upregulated in Liver Tissues of HFD-Fed Mice after Treatment with Aqueous Bupleuri Radix Extract

#### 3.5.1. Histomorphology of Liver Tissues

**Figure 5:**
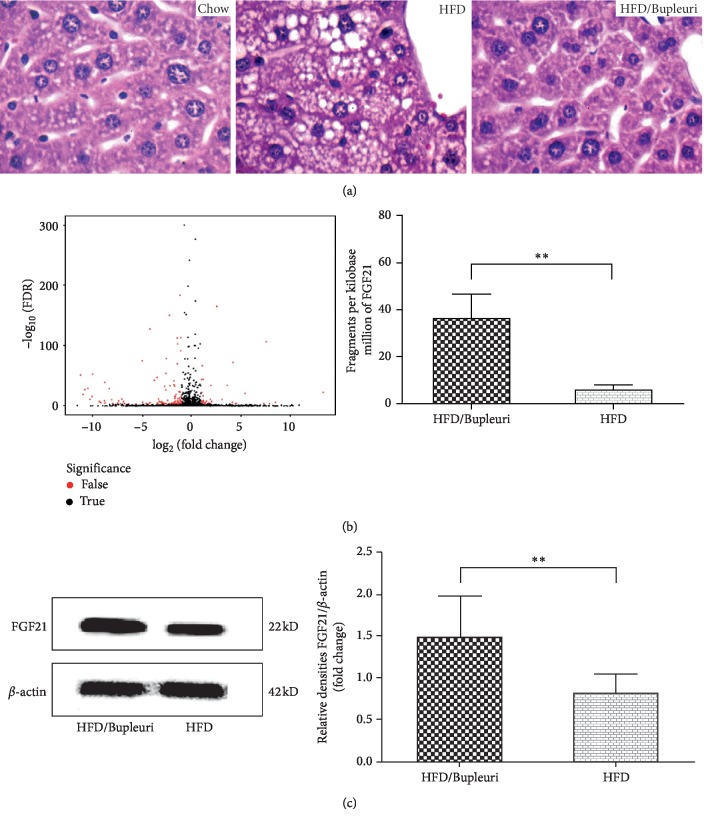
Upregulation of FGF21 was observed in the livers of HFD-fed mice treated with aqueous Bupleuri Radix extract. (a) Liver tissue morphology. Representative photomicrographs (magnification ×40) with hematoxylin-eosin stain. The administration group showed noticeable changes in steatosis and hepatocyte ballooning compared to the HFD group. (b) Results of RNA-sequencing analysis. The left panel was the volcano map of differentially expressed genes between the HFD and HFD/Bupleuri groups, FDR ≤ 0.001, log2 (*Y*/*X*) ≥ 1. Digging in the volcano map, FGF21 was found highly expressed in the livers of the HFD/Bupleuri group as shown in the right panel. (c) Western blotting test demonstrated the upregulation of FGF21 in the livers of the HFD/Bupleuri group. Liver tissues were collected and lysed with 2 × SDS sample buffer. *β*-Actin was used to show the same loading of the liver tissue. ^*∗∗*^*p* < 0.01, compared to the HFD group.

Compared to the chow mice, disorganized lobules, steatosis, fat vesicles, and unclear boundary were observed in livers of HFD mice, whereas liver cells of HFD/Bupleuri mice were intact and regular, and no obvious fatty degeneration and necrosis were found (Figure 5(a)), indicating aqueous Bupleuri Radix extract ameliorated hepatic steatosis induced by high-fat diet.

#### 3.5.2. RNA-Sequencing Analysis

RSEM software packages were utilised for quantifying gene and isoform abundances, described as fragments per kilobase of exon model per million fragments mapped (FPKM), from paired-end RNA-sequencing data. Principal component analysis, coexpressed gene and correlation stat, condition specificity analysis, and screening of differentially expressed genes (DEG) were carried out for data manipulation of RNA sequencing. For statistical analysis of DEG, *p* values from the *t*-tests of FPKM were converted to FDR-adjusted *Q* values. Only those transcripts that have a *Q* value lower than the given FDR (≤0.001) were further analysed, and in output files, all DEG values were FDR-corrected, *p* < 0.001 as shown in the volcano plot (Figure 5(b), red dots represent significant DEG). To elucidate the underlying target of Bupleuri Radix aqueous extract on HDL uptake from these differentially expressed genes between HFD and HFD/Bupleuri mice, cluster analysis, Gene Ontology classification, and pathway enrichment were utilized to assign gene changes to different functional categories. Seeking in category of metabolism, an extremely higher expression of FGF21 in livers of HFD/Bupleuri mice was found than in HFD mice (Figure 5(b), *p* < 0.0001).

#### 3.5.3. Western Blotting Validation

Recent genetic data demonstrated a strong causal role for FGF21 in the aetiology of metabolic diseases in humans. We therefore prospectively adapted western blotting to testify the upregulation of FGF21 expression in the liver of HFD-fed mice after treatment with Bupleuri Radix aqueous extract. Gray-scale and statistical analyses revealed that FGF21 levels in the livers of HFD/Bupleuri mice were significantly higher than HFD-mice (Figure 5(c)), verifying aqueous Bupleuri Radix extract elevated FGF21 expression level in the liver of HFD-fed mice.

## 4. Conclusions

A variety of studies [16, 17] have claimed that primary saikosaponins (SSa, SSd, SSc, etc.) have unstable chemical structure and immensely switch into secondary saikosaponins (SSb_1_, SSb_2_, SSh, etc.) when Bupleuri Radix is extracted in boiling water, while they are stable in extraction with methanol. Technically, secondary saikosaponins are not the secondary metabolites occurring in raw plants, but they mainly occur during aqueous extraction process and their chemical structures are quite distinguishable from primary saikosaponins. Our study tends to indicate that secondary saikosaponins are the bioactive components in Bupleuri Radix and contribute dramatically to HDL-uptake capacity of HepG2 cells, whereas primary saikosaponins exert inhibiting hepatic uptake of HDL.

We also tried to reveal the mechanism of how the aqueous extract of Bupleuri Radix elevated hepatic uptake of HDL via RNA sequencing in this study. We found the gene of FGF21 was highly expressed in the livers of mice exposed to aqueous extract in comparison with the control, and western blotting analysis testified this result. FGF21 is primarily produced and secreted by the liver as a hepatokine, and as a novel metabolic regulator, it has been extensively reviewed [18, 19] to play a vital role in the regulation of energy homeostasis in both preclinical studies and in man since its initial isolation from the liver in 2000. Recent emerging clinical evidence [20–22] has shown that serum FGF21 levels in obese subjects were remarkably higher than in healthy individuals, and significant negative relationships between serum FGF21 concentration and HDL-cholesterol level were observed in these studies. Administering FGF21 analogue significantly increases HDL-C and reduces triglycerides in obese human subjects with type 2 diabetes [23] or hypertriglyceridaemia [24]. We therefore prospectively conjecture aqueous extract of Bupleuri Radix promotes HDL uptake via targeting FGF21, but the detailed cellular and molecular mechanisms by which FGF21 mediates downstream signaling pathways are likely to be complex and need to be investigated in the next step.

## Data Availability

The CCK-8 assay, FACS, RNA sequence, and western blotting data used to support the findings of this study are available from the corresponding author upon request. The HPLC chromatograms, FACS histograms, micrographs of HDL uptake, and histologic data used to support the findings of this study are included within the article.

## Abbreviations

HDL: High-density lipoproteins
SSb2: Saikosaponin b2
SSb1: Saikosaponin b1
SSa: Saikosaponin a
SSd: Saikosaponin d
SSc: Saikosaponin c
DiI: Labeled with the fluorescent probe 1,1′-dioctadecyl-3,3,3′, 3′-tetramethylindocarbocyanine perchlorate
HPLC-DAD: High-performance liquid chromatography with photodiode array detection
FPKM: Fragments per kilobase of exon model per million fragments mapped
DEG: Differentially expressed genes
HFD: High-fat diet.
